# Systematic analyses of glutamine and glutamate metabolisms across different cancer types

**DOI:** 10.1186/s40880-017-0255-y

**Published:** 2017-11-07

**Authors:** Yuan Tian, Wei Du, Sha Cao, Yue Wu, Ning Dong, Yan Wang, Ying Xu

**Affiliations:** 1 College of Computer Science and Technology, Jilin University, Changchun, 130012 Jilin P. R. China; 2 The First Hospital, Jilin University, Changchun, 130012 Jilin P. R. China; 3 College of Public Health, Jilin University, Changchun, 130012 Jilin P. R. China; 4 Computational Systems Biology Lab, Department of Biochemistry and Molecular Biology and Institute of Bioinformatics, University of Georgia, 120 E Green St, Athens, GA 30602 USA

**Keywords:** Glutamine metabolism, Glutamate metabolism, Nucleotide synthesis, Lipid synthesis, Uridine diphosphate *N*-acetylglucosamine (UDP-GlcNAc) metabolism

## Abstract

**Background:**

Glutamine and glutamate are known to play important roles in cancer biology. However, no detailed information is available in terms of their levels of involvement in various biological processes across different cancer types, whereas such knowledge could be critical for understanding the distinct characteristics of different cancer types. Our computational study aimed to examine the functional roles of glutamine and glutamate across different cancer types.

**Methods:**

We conducted a comparative analysis of gene expression data of cancer tissues versus normal control tissues of 11 cancer types to understand glutamine and glutamate metabolisms in cancer. Specifically, we developed a linear regression model to assess differential contributions by glutamine and/or glutamate to each of seven biological processes in cancer versus control tissues.

**Results:**

While our computational predictions were consistent with some of the previous observations, multiple novel predictions were made: (1) glutamine is generally not involved in purine synthesis in cancer except for breast cancer, and is similarly not involved in pyridine synthesis except for kidney cancer; (2) glutamine is generally not involved in ATP production in cancer; (3) glutamine’s contribution to nucleotide synthesis is minimal if any in cancer; (4) glutamine is not involved in asparagine synthesis in cancer except for bladder and lung cancers; and (5) glutamate does not contribute to serine synthesis except for bladder cancer.

**Conclusions:**

We comprehensively predicted the roles of glutamine and glutamate metabolisms in selected metabolic pathways in cancer tissues versus control tissues, which may lead to novel approaches to therapeutic development targeted at glutamine and/or glutamate metabolism. However, our predictions need further functional validation.

## Background

Glutamine is the most abundant type of amino acid in human blood circulation [1, 2]. Its utilization in cancer cells is considerably increased compared with that in normal human tissue cells [3]. Substantial research efforts have been invested to study the utilization of glutamine in cancer including its roles in protein and nucleotide synthesis [4], glutaminolysis for energy generation [5, 6], conversion to other amino acids such as serine that is needed for nicotinamide adenine dinucleotide phosphate (NADPH) and purine synthesis [7, 8], glutathione (GSH) synthesis for anti-oxidation [9–11], and uridine diphosphate *N*-acetylglucosamine (UDP-GlcNAc) synthesis for *O*-glycan [12] and heparan sulfate production [13]. Glutamate has also been found to be utilized by cancer cells. Previous studies have found that glutamate is involved in glutaminolysis [14] and GSH synthesis [15] in multiple types of cancer. While numerous studies on elucidation of the functional roles of glutamine and glutamate in cancer have been published [16–19], no comparative analyses of their functions across different cancer types have been published, to the best of our knowledge.

We conducted here a computational analysis of gene expression data of cancer tissues versus normal control tissues of 11 types of human cancer based on The Cancer Genome Atlas (TCGA) gene expression data [20, 21], focusing on glutamine and glutamate metabolisms. We addressed the following four questions through our analyses. (1) Do cancers generally have increased influx of glutamine and/or glutamate? (2) Are glutamine and glutamate metabolisms increased in each type of cancer under consideration? (3) In what major biological processes are glutamine and glutamate involved across different types of cancer? (4) For types of cancer that do not involve glutamine and/or glutamate in some of these biological processes, how are the processes accomplished?

## Methods

### Dataset

Gene expression data measured by RNA-seq of 34 types of human cancer were downloaded from the TCGA database [22], and only those types of cancer with at least 10 cancerous and 10 control samples were kept.

### Identification of differentially expressed genes

A gene was deemed to be up- or down-regulated if the fold change between the average expression level of the gene in cancer samples and that in control samples is larger than 1.5 or smaller than − 1.5, with a *P* value no more than 0.05 measured using the limma *t* test [23].

### Estimating expression level of metabolic process

Given a metabolic process *m*, its expression level was estimated using the expression levels of their rate-limiting enzyme or transporter genes. In cases when multiple enzymes or transporters were associated with *m*, a principal component analysis was applied to the expression matrix of these enzymes or transporters [24], and the first principal component was obtained as the one-dimension representative of these genes and was used as the expression level of *m*, with rationale of dimension deduction.

### Assessing statistical contribution of substrate towards synthesis of product

For a product metabolite *p*, we identified whether contributions from potential reactant metabolite(s) may differ in cancer versus controls.

We built a multiple group regression model [25] and used it to check whether differences exist between cancer and controls in reactant metabolites $r_{1} , \ldots ,r_{M}$ contributing to product* p*:

$${\vec{\mathbf{y}}}_{p} = \beta_{0} + \alpha_{0} :{\mathbf{ID}} + \beta_{1} {\vec{\mathbf{x}}}_{1} + \cdots + \beta_{M} {\vec{\mathbf{x}}}_{M} + \alpha_{1} {\vec{\mathbf{x}}}_{1} :{\mathbf{ID}} + \cdots + \alpha_{M} {\vec{\mathbf{x}}}_{M} :{\mathbf{ID}},$$

where $\vec{\varvec{y}}$ is the observed level of product metabolite *p* across all samples of both cancer and controls; $\varvec{ ID}$ is an index variable denoting whether observations are from cancer (= 1) or control (= 0) samples; $\vec{\varvec{x}}_{1} , \ldots ,\vec{\varvec{x}}_{M}$ are the observed levels of reactant metabolites $r_{1} , \ldots ,r_{M}$ via synthesis and/or up-take (for transporter genes) across all samples; $\vec{\varvec{x}}_{1} :\varvec{ID}, \ldots ,\vec{\varvec{x}}_{M} :\varvec{ID}$ denote the interactions between the index variables $\varvec{ ID}$ and $\vec{\varvec{x}}_{1} , \ldots ,\vec{\varvec{x}}_{M}$; and $\beta_{0} , \beta_{1} , \ldots ,\beta_{M} ,\alpha_{0} ,\alpha_{1} , \ldots ,\alpha_{M}$ are regression coefficients. The interaction terms between the index variable and other predictor variables denote the differential contributions of these metabolites to the production of product metabolite *p* in cancer versus control samples. In a fitted regression model, if some interaction terms are found to be statistically significant, the corresponding metabolites are predicted to contribute differently to the production of *p* in cancer versus control tissues. Linear regression model and assessment of the significance of linear terms were done using R software (R Foundation for Statistical Computing, Vienna, Austria).

## Results

### The influxes of glutamine and glutamate are substantially increased in cancer versus control tissues

Eleven types of cancer, including bladder urothelial carcinoma (BLCA), breast invasive carcinoma (BRCA), colon adenocarcinoma (COAD), head and neck squamous cell carcinoma (HNSC), kidney chromophobe (KICH), kidney renal clear cell carcinoma (KIRC), kidney renal papillary cell carcinoma (KIRP), liver hepatocellular carcinoma (LIHC), lung adenocarcinoma (LUAD), prostate adenocarcinoma (PRAD), and thyroid carcinoma (THCA), were finally included in the present study, with details presented in Table 1. We compared gene expression data of cancer versus control tissues of the 11 types of cancer to detect whether cancers generally have increased uptake or synthesis of glutamine and glutamate. Specifically, we examined the expression levels of all the genes that encode importers or synthases for glutamine and glutamate separately, which are summarized in Table 2(A1–4).

**Table 1:** Sample sizes of RNA-seq data for 11 cancer types

Type	Cancer tissue (samples)	Control tissue (samples)
Bladder urothelial carcinoma (BLCA)	182	18
Breast invasive carcinoma (BRCA)	994	106
Colon adenocarcinoma (COAD)	233	21
Head and neck squamous cell carcinoma (HNSC)	303	37
Kidney chromophobe (KICH)	66	25
Kidney renal clear cell carcinoma (KIRC)	480	71
Kidney renal papillary cell carcinoma (KIRP)	141	30
Liver hepatocellular carcinoma (LIHC)	134	49
Lung adenocarcinoma (LUAD)	470	58
Prostate adenocarcinoma (PRAD)	195	45
Thyroid carcinoma (THCA)	494	58

**Table 2:** Genes involved in biological processes related to metabolisms of glutamine and glutamate

Process^a^	Gene(s)
A1. Glutamine uptake	SLC1A5, SLC38A1, SLC38A2, SLC38A3, SLC38A5
A2. Glutamine synthesis	GLUL
A3. Glutamate uptake	SLC1A1, SLC1A2, SLC1A3, SLC1A6, SLC1A7
A4. Glutamate synthesis	GLS, GLS2, PFAS, GMPS, CAD, CTPS, CTPS2
B1. Purine de novo synthesis from glutamine	PPAT
B2. Purine synthesis by salvage from adenosine	APRT, ADA, ADK
B3. Purine synthesis by salvage from guanosine	APRT, HPRT1
B4. Purine synthesis by salvage from inosine	HPRT1
B5. Adenosine uptake	SLC28A1, SLC28A2, SLC28A3, SLC29A1, SLC29A2, SLC29A3, SLC29A4
B6. Guanosine uptake	SLC28A2, SLC28A3, SLC29A1, SLC29A2, SLC29A3
B7. Inosine uptake	SLC28A2, SLC28A3
C1. Pyrimidine de novo synthesis from glutamine	CAD
C2. Pyrimidine synthesis by salvage from cytidine	UCK1, UCK2, UCKl1
C3. Pyrimidine synthesis by salvage from uridine	UCK1, UCK2, UCKl1, UPP1, UPP2, UPRT
C4. Cytidine uptake	SLC28A1, SLC28A2, SLC28A3, SLC29A1, SLC29A2, SLC29A3
C5. Uridine uptake	SLC28A1, SLC28A2, SLC28A3, SLC29A1, SLC29A2, SLC29A3
D1. Glycolysis	HK1, HK2, HK3, HKDC1, GCK, PFKL, PFKM, PFKP, PKLR, PKM2
D2. Oxidative phosphorylation	ATP5A1, ATP5B, ATP5C1, ATP5D, ATP5E, ATP5F1, ATP5G1, ATP5G2, ATP5G3, ATP5H, ATP5I, ATP5 J, ATP5J2, ATP5L, ATP5O
D3. Glucose uptake	SLC2A1, SLC2A2, SLC2A3, SLC2A4, SLC2A5, SLC2A6, SLC2A8, SLC2A9, SLC2A10, SLC2A12, SLC2A14, SLC5A2, SLC5A1, SLC5A4, SLC5A9, SLC5A10
E1. Lipid synthesis	ACACA, ACACB, FASN
E2. Lipid uptake	FABP1, FABP2, FABP3, FABP4, FABP5, FABP6, FABP7, FABP12, APOBR, CD36, CXCL16, ILDR1, LDLR, LRP1, LRP10, LRP12, LRP2, LRP6, LRP8, OLR1, SCARB1, STAB 1, STAB 2, VLDLR
F1. UDP-GlcNAc synthesis from glutamine	HK1, HK2, HK3, HKDC1, GCK, GFPT1, GFPT2
F2. UDP-GlcNAc synthesis from glucosamine	HK1, HK2, HK3, HKDC1, GNPNAT1, PGM3, UAP1, UAP1L1
F3. Glucosamine uptake	SLC2A2
G1. Asparagine synthesis from glutamine	ASNS
G2. Asparagine uptake	SLC1A5, SLC38A3, SLC38A7
G3. Exchange of asparagine for other amino acids	SLC1A4, SLC7A1, SLC7A5
H1. Proline synthesis from glutamine	ALDH18A1, PYCR1, PYCR2, PYCRL
H2. Proline synthesis from arginine	ARG1, ARG2, OAT, PYCR1, PYCR2, PYCRL
H3. Proline uptake	SLC1A4, SLC6A7, SLC6A15, SLC36A4, SLC36A1, SLC36A2, SLC36A3
H4. Arginine uptake	SLC7A1, SLC7A2, SLC7A3, PQLC2
H5. Arginine synthesis	ASS1
I1. Serine synthesis from glutamine	PHGDH, PSAT1, PSPH
I2. Serine uptake	SLC1A4, SLC1A5, SLC7A1, SLC7A5, SLC7A10, SLC38A7, SERINC1, SERINC2, SERINC3, SERINC4, SERINC5
J1. GSH synthesis from glutamate	GCLC, GCLM, GSS
J2. TXN and catalase synthesis	TXN, TXNRD1, TXNRD2, CAD

^a^The first letter in each item including A–J represents each group of genes that have the same biological process

UDP-GlcNAc, uridine diphosphate *N*-acetylglucosamine; GSH, glutathione; TXN, thioredoxin; SLC1A5, solute carrier family 1 member 5; GLUL, glutamate–ammonia ligase; GLS, glutaminase; PFAS, phosphoribosylformylglycinamidine synthase; GMPS, guanine monophosphate synthase; CAD, carbamoyl-phosphate synthetase 2, aspartate transcarbamylase, and dihydroorotase; CTPS1, CTP synthase 1; PPAT, phosphoribosyl pyrophosphate amidotransferase; APRT, adenine phosphoribosyltransferase; ADA, adenosine deaminase; ADK, adenosine kinase; HPRT1, hypoxanthine phosphoribosyltransferase 1; UCK1, uridine–cytidine kinase 1; UCKl1, uridine–cytidine kinase 1 like 1; UPP1, uridine phosphorylase 1; UPRT, uracil phosphoribosyltransferase homolog; HK1, hexokinase 1; HKDC1, hexokinase domain containing 1; GCK, glucokinase; PFKL, phosphofructokinase, liver type; PFKM, phosphofructokinase, muscle; PKLR, pyruvate kinase, liver and RBC; PKM2, pyruvate kinase, muscle; ATP5A1, ATP synthase, H^+^ transporting, mitochondrial F1 complex, alpha subunit 1, cardiac muscle; ACACA, acetyl-CoA carboxylase alpha; FASN, fatty acid synthase; FABP1, fatty acid binding protein 1; APOBR, apolipoprotein B receptor; CD36, CD36 molecule; CXCL16, C-X-C motif chemokine ligand 16; ILDR1, immunoglobulin like domain containing receptor 1; LDLR, low density lipoprotein receptor; LRP1, LDL receptor related protein 1; OLR1, oxidized low density lipoprotein receptor 1; SCARB1, scavenger receptor class B member 1; STAB 1, stabilin 1; VLDLR, very low density lipoprotein receptor; GFPT1, glutamine-fructose-6-phosphate transaminase 1; GNPNAT1, glucosamine-phosphate *N*-acetyltransferase 1; PGM3, phosphoglucomutase 3; UAP1, UDP-*N*-acetylglucosamine pyrophosphorylase 1; UAP1L1, UDP-*N*-acetylglucosamine pyrophosphorylase 1 like 1; ASNS, asparagine synthetase; ALDH18A1, aldehyde dehydrogenase 18 family member A1; PYCR1, pyrroline-5-carboxylate reductase 1; PYCRL, pyrroline-5-carboxylate reductase-like; ARG1, arginase 1; OAT, ornithine aminotransferase; PQLC2, PQ loop repeat containing 2; ASS1, argininosuccinate synthase 1; PSAT1, phosphoserine aminotransferase 1; PSPH, phosphoserine phosphatase; SERINC1, serine incorporator 1; SGCLC, glutamate–cysteine ligase catalytic subunit; GCLM, glutamate–cysteine ligase modifier subunit; GSS, glutathione synthetase; TXNRD1, thioredoxin reductase 1

**Fig. 1:**
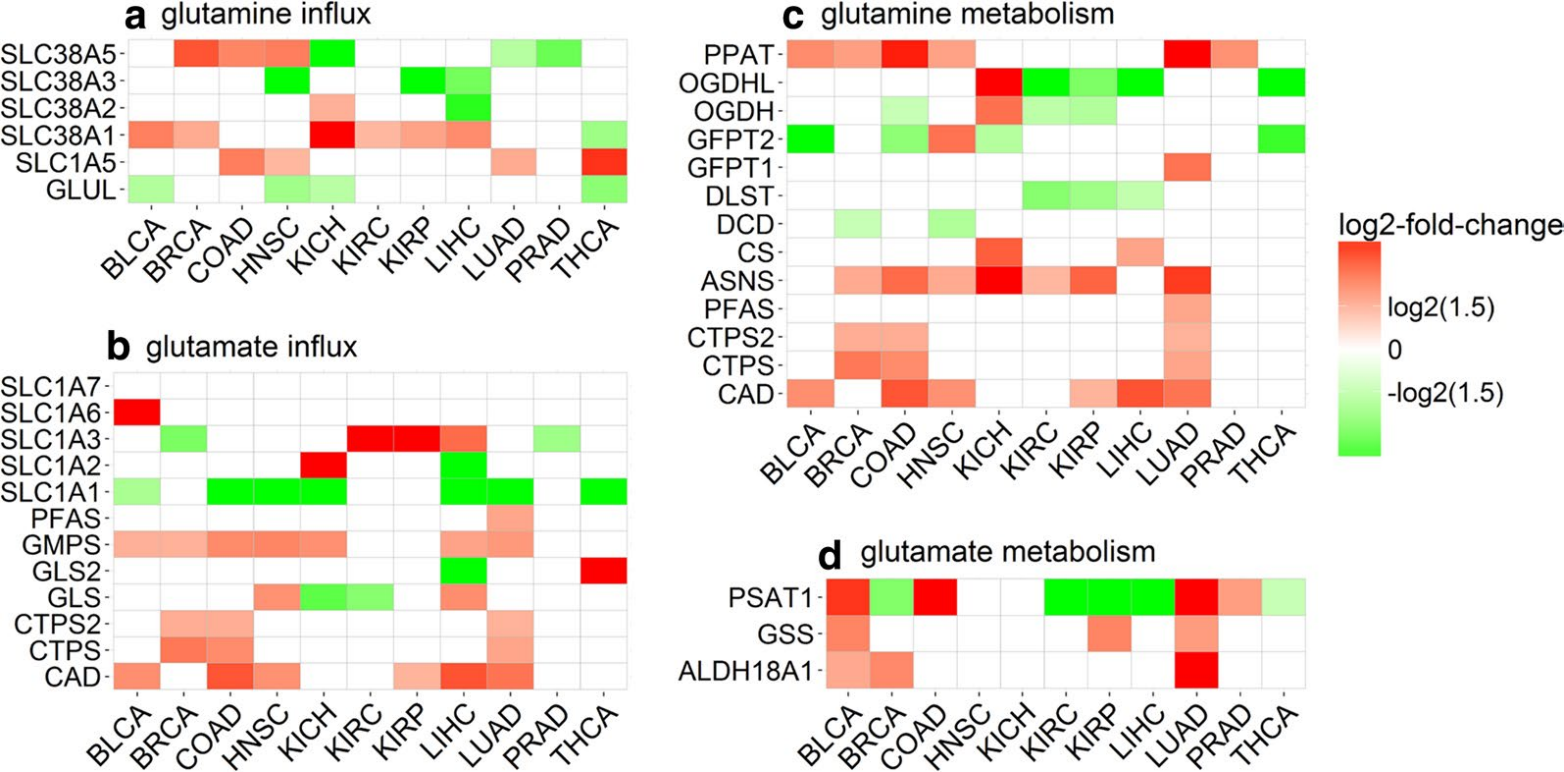
Differential expression of importer and synthase genes for **a** glutamine and **b** glutamate influx, and metabolisms of **c** glutamine and **d** glutamate in 11 types of cancer. BLCA, bladder urothelial carcinoma; BRCA, breast invasive carcinoma; COAD, colon adenocarcinoma; HNSC, head and neck squamous cell carcinoma; KICH, kidney chromophobe; KIRC, kidney renal clear cell carcinoma; KIRP, kidney renal papillary cell carcinoma; LIHC, liver hepatocellular carcinoma; LUAD, lung adenocarcinoma; PRAD, prostate adenocarcinoma; THCA, thyroid carcinoma; SLC1A5, solute carrier family 1 member 5; GLUL, glutamate–ammonia ligase; GLS, glutaminase; PFAS, phosphoribosylformylglycinamidine synthase; GMPS, guanine monophosphate synthase; CAD, carbamoyl-phosphate synthetase 2, aspartate transcarbamylase, and dihydroorotase; CTPS, CTP synthase 1; PPAT, phosphoribosyl pyrophosphate amidotransferase; OGDHL, oxoglutarate dehydrogenase-like; OGDH, oxoglutarate dehydrogenase; GFPT1, glutamine-fructose-6-phosphate transaminase 1; DSLT, dihydrolipoamide S-succinyltransferase; DCD, dermcidin; CS, citrate synthase; ASNS, asparagine synthetase; PSAT1, phosphoserine aminotransferase 1; GSS, glutathione synthetase; ALDH18A1, aldehyde dehydrogenase 18 family member A1

Differential expression analyses were conducted on these genes with the detailed results shown in Fig. 1a, b. We noted that among the five known glutamine importers, solute carrier family 1 member 5 (SLC1A5) [26–30] and solute carrier family 38 member 1 (SLC38A1) [31–34] were widely studied and are up-regulated in four and six types of cancer, respectively; two less studied importers, solute carrier family 38 member 2 (SLC38A2) [35] and solute carrier family 38 member 5 (SLC38A5) [36], are overexpressed in one and three types of cancer, respectively; and solute carrier family 38 member 3 (SLC38A3) [37] is not up-regulated in any cancer (Fig. 1a). In addition, glutamate–ammonia ligase (GLUL) is not overexpressed in any type of cancer under study, hence indicating that glutamine is not synthesized from glutamate in cancer in general. We also discovered that SLC38A1 is overexpressed in BLCA; SLC1A5 and SLC38A5 in HNSC; SLC38A1 and SLC38A2 in KIRC; SLC38A1 in KIRP; and SLC1A5 in THCA. In addition, we found that the uptake of glutamine is increased by up-regulated SLC38A1 and SLC38A5 in BRCA, where such observation with SLC38A1 was previously reported [32]; by up-regulated SLC1A5 and SLC38A5 in COAD, where such observation with SLC1A5 was previously reported [30]; and by up-regulated SLC38A1 instead of SLC1A5 in KIRP as previously reported [27]. The increased influx of glutamate is due to increased conversion from glutamine and increased uptake in at least six types of cancer as shown in Fig. 1b.

Overall, we found that 10 of the 11 types of cancer have increased influxes of both glutamine and glutamate, and PRAD is the only type of cancer (under consideration) without increased utilization of glutamine or glutamate, compared with the normal controls.

### Basic metabolisms of glutamine and glutamate are up-regulated in cancer

To identify whether glutamine and glutamate metabolisms are increased in each type of cancer under consideration, we examined the expression levels of 13 and 3 genes involved in the basic glutamine and glutamate metabolisms, respectively, referred to as the glutamine and glutamate metabolic genes, consisting of rate-limiting enzyme genes in glutaminolysis and genes for catalyzing reactions that directly involve glutamine or glutamate.

The differential expression of the above genes is shown in Fig. 1c, d. Specifically, among the 11 types of cancer, we found that LUAD has the highest up-regulated glutamine metabolism, which agrees with the results of a published study [38]. COAD, KICH, HNSC, and BRCA also have substantial up-regulation in the glutamine metabolism, indicating an important role of glutamine metabolism in these cancers as reported in the study [39]. We also found that LUAD has the highest up-regulation in the glutamate metabolism, whereas KIRC, LIHC, and THCA have the lowest.

**Fig. 2:**
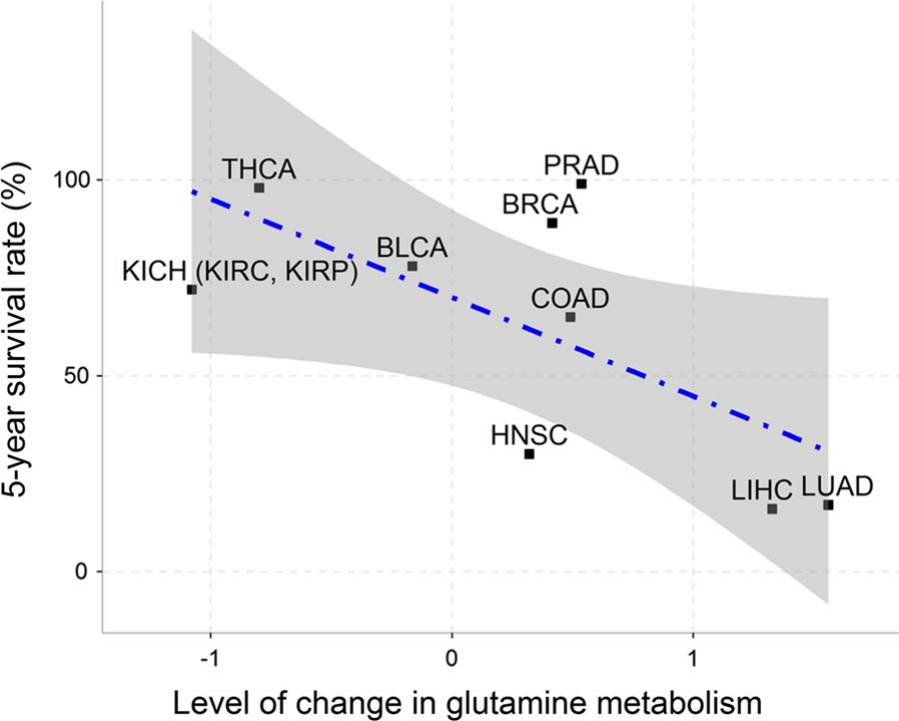
Significant negative correlation between the increased glutamine metabolism and 5-year survival rate across the 11 types of cancer. The *X* axis represents the level of change in glutamine metabolism, which is the difference between the levels of glutamine metabolism in cancer versus control tissues; the *Y* axis represents the 5-year survival rate of cancer. The average level of change in glutamine metabolism across three subtypes of the kidney cancer (KICH, KIRC, and KIRP) is used because there are no 5-year survival data for the individual subtypes. BLCA, bladder urothelial carcinoma; BRCA, breast invasive carcinoma; COAD, colon adenocarcinoma; HNSC, head and neck squamous cell carcinoma; KICH, kidney chromophobe; KIRC, kidney renal clear cell carcinoma; KIRP, kidney renal papillary cell carcinoma; LIHC, liver hepatocellular carcinoma; LUAD, lung adenocarcinoma; PRAD, prostate adenocarcinoma; THCA, thyroid carcinoma

We noted a negative correlation between the level of change in glutamine metabolism and the average 5-year survival rate across the 11 types of cancer [40], with a corresponding *r* of − 0.604 and a *P* value of 0.049 (Fig. 2), suggesting that the expression levels of glutamine metabolic genes can potentially be used as an indicator for the survival rate of a cancer patient. Specifically, level of change in glutamine metabolism represents the difference between expression levels of glutamine metabolism in cancer versus control tissues.

### Biological functions that involve glutamine and glutamate

A systematic review of the literature [16–19] suggests that glutamine and glutamate are involved in seven pathways in cancer (Table 3). We conducted systematic analyses coupled with modeling to identify (i) what major biological processes glutamine or glutamate is involved in across different types of cancer, respectively; and (ii) for cancer types that do not involve glutamine or glutamate in some of these biological processes, what might have been used instead.

**Table 3:** Biological pathways with increased glutamine or glutamate influx across 11 types of cancer

Pathway	Substrate → product	Cancer type(s)
Purine/pyrimidine metabolisms	Glutamine → nucleotides	BRCA, KIRC
Biomass production	Glutamine → biomass	PRAD, THCA
UDP-GlcNAc metabolism	Glutamine → UDP-GlcNAc	BRCA, PRAD
Asparagine metabolism	Glutamine → asparagine	BLCA, LUAD
Proline metabolism	Glutamine → proline	PRAD
Serine metabolism	Glutamate → serine	BLCA
GSH metabolism	Glutamate → GSH	BRCA, KICH, LUAD

UDP-GlcNAc, uridine diphosphate *N*-acetylglucosamine; GSH, glutathione

#### Glutamine is involved in purine and pyrimidine biosynthesis in cancer

**Fig. 3:**
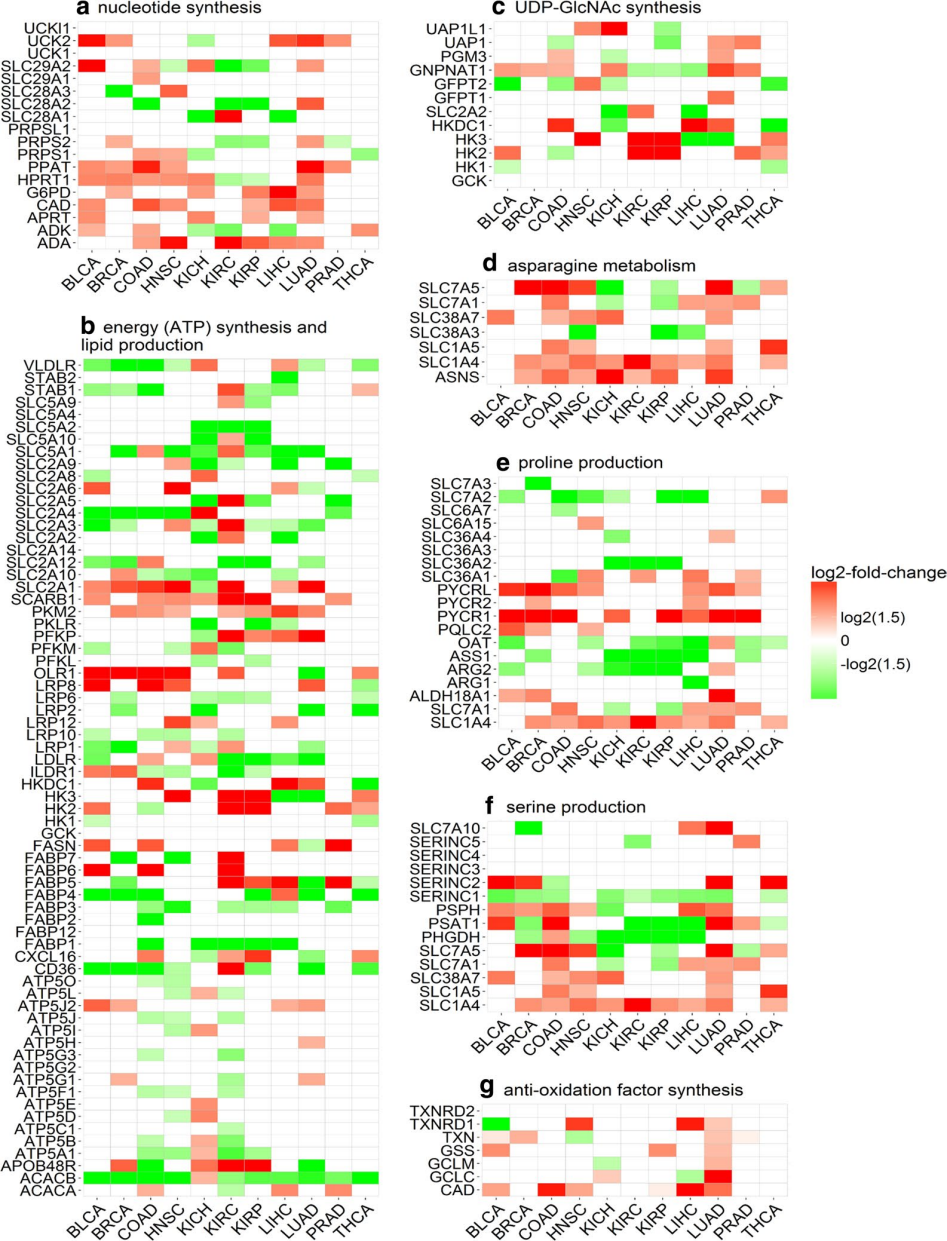
Differential expression of rate-limiting enzyme genes and transporter genes involved in **a** nucleotide synthesis, **b** energy (ATP) synthesis and lipid production, **c** uridine diphosphate *N*-acetylglucosamine (UDP-GlcNAc) synthesis, **d** asparagine metabolism, **e** proline production, **f** serine production, and **g** anti-oxidation factor synthesis. BLCA, bladder urothelial carcinoma; BRCA, breast invasive carcinoma; COAD, colon adenocarcinoma; HNSC, head and neck squamous cell carcinoma; KICH, kidney chromophobe; KIRC, kidney renal clear cell carcinoma; KIRP, kidney renal papillary cell carcinoma; LIHC, liver hepatocellular carcinoma; LUAD, lung adenocarcinoma; PRAD, prostate adenocarcinoma; THCA, thyroid carcinoma; PPAT, phosphoribosyl pyrophosphate amidotransferase; APRT, adenine phosphoribosyltransferase; ADA, adenosine deaminase; ADK, adenosine kinase; HPRT1, hypoxanthine phosphoribosyltransferase 1; SLC28A1, solute carrier family 28 member 1; CAD, carbamoyl-phosphate synthetase 2, aspartate transcarbamylase, and dihydroorotase; UCK1, uridine–cytidine kinase 1; UCKl1, uridine–cytidine kinase 1 like 1; UPP1, uridine phosphorylase 1; UPRT, uracil phosphoribosyltransferase homolog; HK1, hexokinase 1; HKDC1, hexokinase domain containing 1; GCK, glucokinase; PFKL, phosphofructokinase, liver type; PFKM, phosphofructokinase, muscle; PKLR, pyruvate kinase, liver and RBC; PKM2, pyruvate kinase, muscle; ATP5A1, ATP synthase, H^+^ transporting, mitochondrial F1 complex, alpha subunit 1, cardiac muscle; SLC2A1, solute carrier family 2 member 1; ACACA, acetyl-CoA carboxylase alpha; ACACB, acetyl-CoA carboxylase beta; FASN, fatty acid synthase; FABP1, fatty acid binding protein 1; APOBR, apolipoprotein B receptor; CD36, CD36 molecule; CXCL16, C-X-C motif chemokine ligand 16; ILDR1, immunoglobulin like domain containing receptor 1; LDLR, low density lipoprotein receptor; LRP1, LDL receptor related protein 1; OLR1, oxidized low density lipoprotein receptor 1; SCARB1, scavenger receptor class B member 1; STAB 1, stabilin 1; STAB 2, stabilin 2; VLDLR, very low density lipoprotein receptor; GFPT1, glutamine-fructose-6-phosphate transaminase 1; GNPNAT1, glucosamine-phosphate *N*-acetyltransferase 1; PGM3, phosphoglucomutase 3; UAP1, UDP-*N*-acetylglucosamine pyrophosphorylase 1; UAP1L1, UDP-*N*-acetylglucosamine pyrophosphorylase 1 like 1; ASNS, asparagine synthetase; ALDH18A1, aldehyde dehydrogenase 18 family member A1; PYCR1, pyrroline-5-carboxylate reductase 1; ARG1, arginase 1; OAT, ornithine aminotransferase; PQLC2, PQ loop repeat containing 2; ASS1, argininosuccinate synthase 1; PSAT1, phosphoserine aminotransferase 1; PSPH, phosphoserine phosphatase; SERINC1, serine incorporator 1; GCLC, glutamate–cysteine ligase catalytic subunit; GCLM, glutamate–cysteine ligase modifier subunit; GSS, glutathione synthetase; TXN, thioredoxin; TXNRD1, thioredoxin reductase 1

It is well established that glutamine is involved in nucleotide synthesis in cancer [16, 19]. Four processes are known for purine synthesis, namely (i) de novo synthesis from glutamine and purine synthesis by salvage from (ii) adenosine, (iii) guanosine, and (iv) inosine. The rate-limiting enzyme genes for each process were used in our analysis, along with the transporter and synthase genes for each metabolite (Table 2A1–2, B1–7) and their differential expressions in cancer versus controls (Figs. 1a, 3a).

**Fig. 4:**
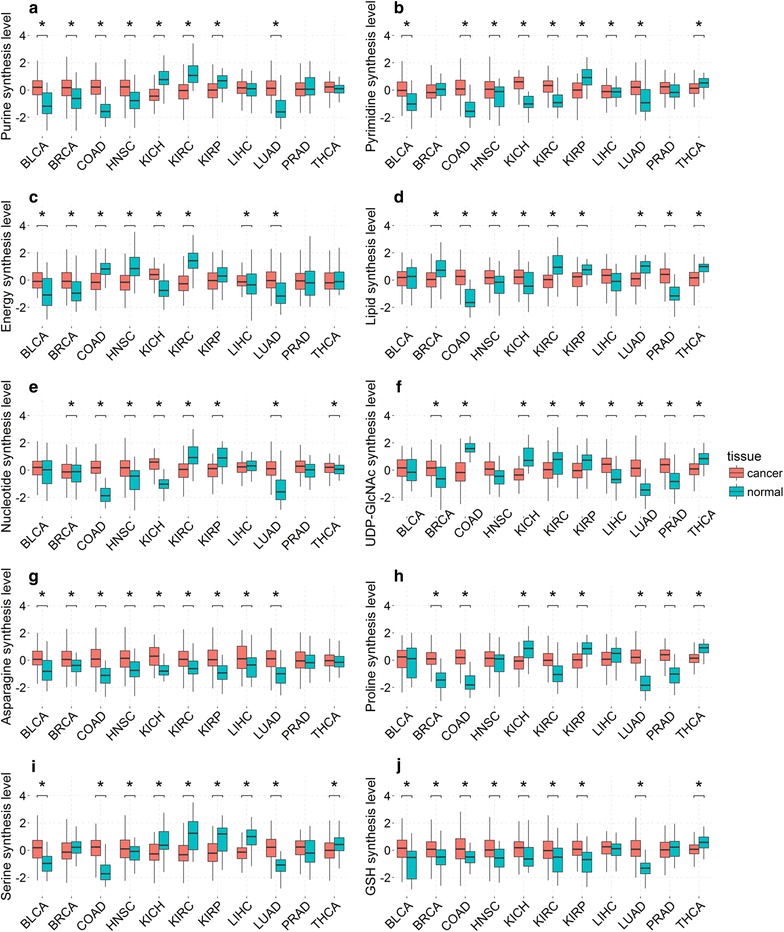
The levels of **a** purine synthesis, **b** pyrimidine synthesis, **c** energy (ATP) synthesis, **d** lipid synthesis, **e** nucleotide synthesis, **f** UDP-GlcNAc synthesis, **g** asparagine synthesis, **h** proline synthesis, **i** serine synthesis, and **j** GSH synthesis in cancer (red) versus control tissues (blue) in 11 types of cancer. In each panel, the *X* axis represents the names of the 11 cancer types, the *Y* axis is for the expression level, and * represents the *P* value no more than 0.05 analyzed with the *t* test. BLCA, bladder urothelial carcinoma; BRCA, breast invasive carcinoma; COAD, colon adenocarcinoma; HNSC, head and neck squamous cell carcinoma; KICH, kidney chromophobe; KIRC, kidney renal clear cell carcinoma; KIRP, kidney renal papillary cell carcinoma; LIHC, liver hepatocellular carcinoma; LUAD, lung adenocarcinoma; PRAD, prostate adenocarcinoma; THCA, thyroid carcinoma

**Fig. 5:**
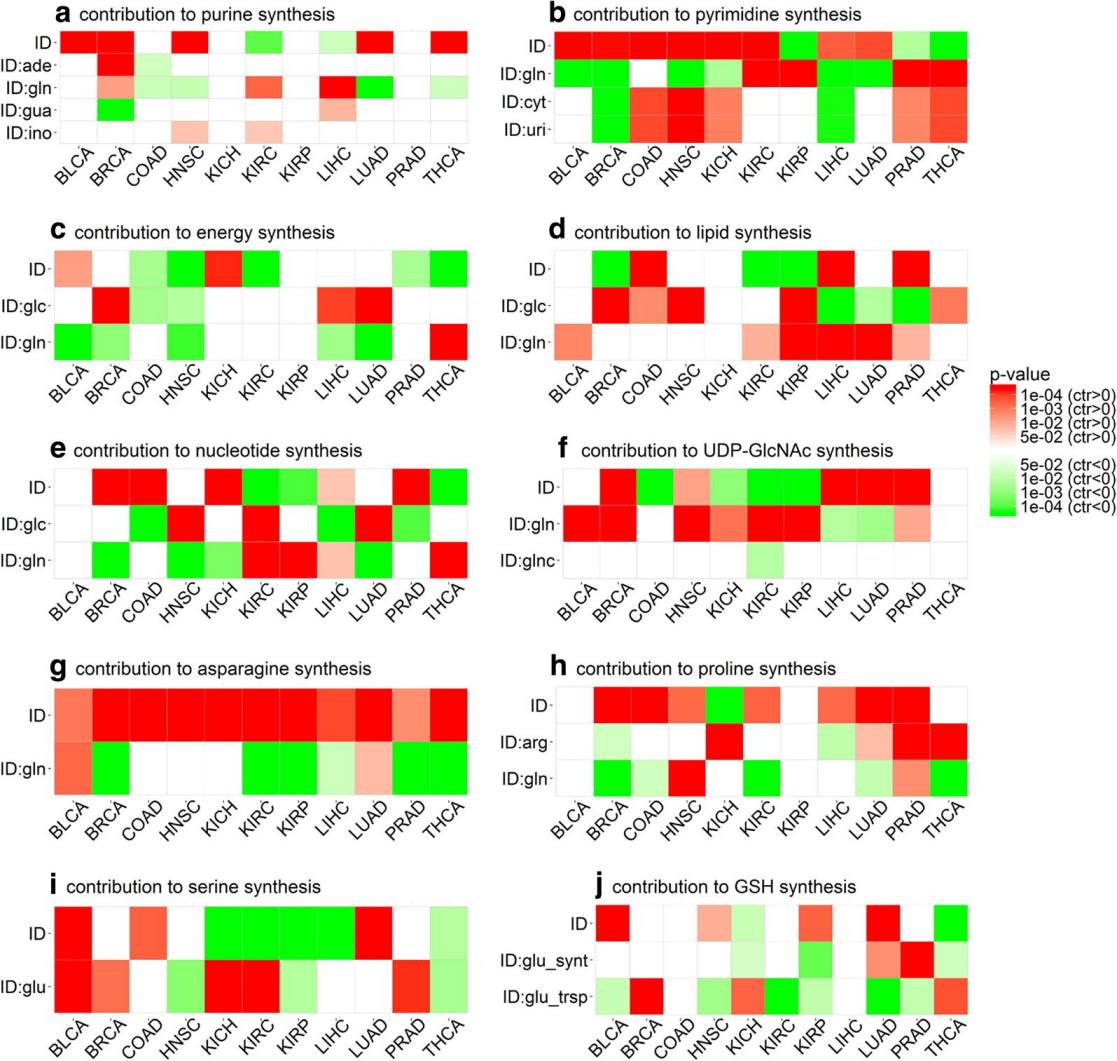
The estimated level of statistical contribution: **a** to purine synthesis from glutamine (gln), adenosine (ade), guanosine (gua) and inosine (ino); **b** pyrimidine synthesis from glutamine, cytidine (cyt) and uridine (uri); **c** energy synthesis from glutamine and glucose (glc); **d** lipid synthesis from glutamine and glucose; **e** nucleotide synthesis from glutamine and glucose; **f** UDP-GlcNAc synthesis from glutamine and glucosamine (glnc); **g** asparagine synthesis from glutamine; **h** proline synthesis from glutamine and arginine (arg); **i** serine synthesis from glutamate (glu); **j** GSH synthesis from glutamate synthesis (glu_synt), and transport (glu_trsp). In each panel, *ID* is a variable for assessing whether the expression level of a response pathway in the linear model differs significantly in cancer and controls, whereas *ID:X* is the estimated contribution of factor *X* to the response pathway, where the color represents if a factor has increased (red) or decreased (green) contribution in cancer and controls. BLCA, bladder urothelial carcinoma; BRCA, breast invasive carcinoma; COAD, colon adenocarcinoma; HNSC, head and neck squamous cell carcinoma; KICH, kidney chromophobe; KIRC, kidney renal clear cell carcinoma; KIRP, kidney renal papillary cell carcinoma; LIHC, liver hepatocellular carcinoma; LUAD, lung adenocarcinoma; PRAD, prostate adenocarcinoma; THCA, thyroid carcinoma

We used the expression levels of the rate-limiting enzyme genes in each of the four purine synthesis pathways to examine the differential expression levels of these pathways in cancer versus controls (Fig. 4a). We then developed a linear regression model based on expression levels of these genes to estimate the differential contributions of glutamine, adenosine, guanosine, and inosine towards purine synthesis in cancer versus control tissues. We noted that purine synthesis is substantially up-regulated in BLCA, BRCA, COAD, HNSC, and LUAD. Among these, BLCA has no consistent patterns in terms of increased contributions by any of the four metabolites; BRCA has increased contributions by both glutamine and adenosine; COAD has no consistent patterns in terms of increased contributions by any but has decreased contributions by glutamine and adenosine; HNSC has increased contribution by inosine and decreased contribution by glutamine; and LUAD has no consistent patterns in terms of increased contributions by any but has decreased contribution by glutamine (Fig. 5a). Overall, there is no consistent pattern across these types of cancer in terms of which of the four metabolites have increased contributions.

A similar analysis was conducted on statistical contribution of glutamine to the synthesis of pyrimidine. The pyrimidine synthesis can be done through one of the following three pathways: (i) pyrimidine de novo synthesis from glutamine and pyrimidine synthesis by salvage from (ii) cytidine or (iii) uridine. Rate-limiting enzyme genes involved in these processes, and transporter and synthase genes for glutamine, cytidine and uridine were included in our analysis (Table 2A1–2, C1–5) with their differential expressions in cancer versus control tissues (Figs. 1a, 3a). Similar to the above analysis, a linear regression model was developed using these genes’ expression levels to assess the contributions of glutamine, cytidine, and uridine to pyrimidine synthesis (Figs. 4b, 5b). Overall, pyrimidine synthesis is up-regulated in seven types of cancer: BLCA, COAD, HNSC, KICH, KIRC, LICH, and LUAD. Among these, the contribution by glutamine is decreased in BLCA and LUAD versus controls; the contributions by cytidine and uridine are increased in COAD; the contributions by cytidine and uridine are increased, and that by glutamine is decreased in HNSC and KICH; the contributions by glutamine is increased in KIRC; and the contribution by any of the three metabolites is decreased in LIHC. Again, like in purine synthesis, there are no consistent patterns in terms of increased or decreased contributions by any of the three contributing factors.

#### Glutamine for energy and biomass synthesis in cancer

Glutamine is the second major nutrient of cancer next to glucose [41]. One of its key functions is to produce energy and biomass. We compared its utilization as an energy producer with that of glucose across the 11 types of cancer. Like in the above subsection, rate-limiting enzyme genes involved in energy production (and biomass synthesis) along with the relevant transporter and synthase genes were included in our analyses (Table 2A1–2, B1–4, C1–3, D1–3 and E1–2) with their differential expression levels in cancer versus control tissues given in Figs. 1a and 3b. Five types of cancer, BLCA, BRCA, KICH, LIHC, and LUAD, have increased ATP synthesis (Fig. 4c). In addition, the levels of contributions towards ATP production by glucose and glutamine are summarized in Fig. 5c, from which we found that, in BRCA, LICH, and LUAD, only glucose has an increased contribution; in BLCA, glutamine has a reduced contribution; and in KICH, glutamine and glucose have no consistent patterns of contribution either increased or decreased.

As for biomass production, the level of lipid synthesis was assessed based on the expression levels of genes encoding acetyl-CoA carboxylase alpha (ACACA), acetyl-CoA carboxylase beta (ACACB), and fatty acid synthase (FASN). Again, a linear regression model was employed to assess the levels of contributions by glutamine and glucose to lipid synthesis using the expressions of these genes along with genes for up-take and syntheses of glutamine and glucose (Figs. 1a, 3b). We found that lipid synthesis is up-regulated in four types of cancer: COAD, HNSC, KICH, and PRAD (Fig. 4d). Among them, COAD and HNSC have increased contributions from glucose; KICH has no consistent patterns in terms of increased or decreased contribution from either glutamine or glucose; and PRAD has increased contribution from glutamine (Fig. 5d). In the other seven types of cancer, lipid is predominantly up-taken from extracellular space based on an observation that fatty acid-binding protein and lipoprotein receptor genes are up-regulated in each of these types of cancer (Fig. 3b).

We also assessed the overall level of nucleotide synthesis, namely purine, pyrimidine, and others from glucose and glutamine (Fig. 4e), and their respective contributions (Fig. 5e). We found that nucleotide synthesis is increased in the following six types of cancer: BRCA, COAD, HNSC, KICH, LUAD, and THCA, among which BRCA and KICH involve less glutamine in cancer versus controls; COAD uses less glucose; HNSC and LUAD use more glucose but less glutamine; and THCA uses more glutamine for nucleotide synthesis.

#### Glutamine is used to generate UDP-GlcNAc in cancer

Cancer tends to have substantially increased glycosylation [42, 43]. UDP-GlcNAc is the basic unit for glycosylation, and it can be synthesized with glutamine [39] or glucosamine [44]. We analyzed contributions by glutamine versus glucosamine to the synthesis of UDP-GlcNAc in cancer versus controls. Like before, we used rate-limiting enzyme genes involved in UDP-GlcNAc synthesis and transporter and synthase genes for glutamine and glucosamine (Table 2A1–2, F1–3) in our analysis. The expression data of these genes are shown in Figs. 1a and 3c. The level of UDP-GlcNAc synthesis is increased in four types of cancer: BRCA, LIHC, LUAD, and PRAD (Fig. 4f). We then assessed the levels of contributions by glutamine and glucosamine separately towards UDP-GlcNAc synthesis (Fig. 5f). Among the four types of cancer, BRCA and PRAD have increased contributions by glutamine in UDP-GlcNAc synthesis; LIHC and LUAD have decreased contributions by glutamine; and no cancers use increased glucosamine towards increased UDP-GlcNAc synthesis.

#### Asparagine synthesized from glutamine in cancer

Asparagine serves as a key exchange factor for extracellular amino acids such as serine in support of cancer growth [45]. One way of increasing asparagine quantity is through synthesis from glutamine, and another is through uptake from circulation. We assessed the activities of the relevant exchangers along with the contributions by glutamine and others to the increased activities of the exchangers in cancer versus control tissues. Table 2(G1–3) summarizes the genes encoding the exchangers along with genes for asparagine synthesis/uptake, with their expression levels in cancer versus control tissues given in Fig. 3d. We used asparagine synthetase (ASNS) to assess the levels of asparagine synthesis in the cancer versus control tissues (Fig. 4g) and found that asparagine synthesis is up-regulated in 10 types of cancer (except for PRAD), among which BLCA and LUAD have increased contributions to asparagine from glutamine; BRCA, KIRC, KIRP, LIHC, and THCA have decreased contributions from glutamine; and COAD, HNSC, and KICH have slightly changed contributions from glutamine (Fig. 5g). Six types of cancer have increased up-take of asparagine from circulation, as the transporter genes are up-regulated in each type of cancer (Fig. 3d).

#### Proline synthesized from glutamine in cancer

Proline plays key roles in cancer such as reactive oxygen species (ROS)-based signaling [46], lipid metabolism [47], and collagen biosynthesis [48]. It is known that proline can be converted from glutamine or arginine. In addition, proline can also be up-taken extracellularly. The genes involved in these processes of proline production, along with the transporter and synthase genes for glutamine and arginine, are summarized in Table 2(A1–2 and H1–5). Figures 1a and 3e show the differential expression levels of these genes in cancer versus control tissues. Proline synthesis is increased in five types of cancer: BRCA, COAD, KIRC, LUAD, and PRAD (Fig. 4h). Specifically, BRCA has decreased contributions from both arginine and glutamine; COAD and KIRC have decreased contribution from glutamine; LUAD has increased contribution by arginine and decreased contribution by glutamine; and PRAD has increased contributions by both glutamine and arginine (Fig. 5h). As shown in Fig. 3e, proline transporter genes are overexpressed in 10 types of cancer (except for BLCA), indicating an increased uptake of proline and increased contributions in these types of cancer.

#### Serine synthesis from glutamate in cancer

Serine is an amino acid used for multiple purposes in cancer, including amino acid synthesis [49–52], DNA/RNA methylation [53], and GSH synthesis [54]. One source for serine is conversion from glutamate via the serine biosynthesis pathway, and the other through up-take. All genes involved in serine production are summarized in Table 2(A3–4 and I1–2), and their differential expression levels in cancer versus control tissues are shown in Figs. 1b and 3f. We used these genes to assess the levels of serine synthesis in cancer versus controls (Fig. 4i). Specifically, four types of cancer, BLCA, COAD, HNSC, and LUAD, have increased serine synthesis. We found that BLCA has increased contribution by glutamate in serine synthesis; its contributions in COAD and LUAD are unchanged; and HNSC has decreased contribution from glutamate (Fig. 5i). It was noteworthy that the serine transporter genes are up-regulated in all 11 types of cancer (Fig. 3f), suggesting that serine uptake represents the predominating approach for increased serine level in cancer cells.

#### Glutathione synthesized from glutamate in cancer

Glutathione is the main anti-oxidation factor in human cells. It is synthesized from cysteine, glutamate, and glycine [55]. The rate-limiting enzyme genes for GSH synthesis along with those for other anti-oxidation factor synthesis are given in Table 2(J1–2), and their differential expression levels in cancer versus control tissues are shown in Fig. 3g. We examined the levels of GSH synthesis in cancer versus control tissues (Fig. 4j) and found that GSH synthesis is increased in the following eight types of cancer: BLCA, BRCA, COAD, HNSC, KICH, KIRC, KIRP, and LUAD. Further analysis revealed the levels of contributions from different sources towards GSH synthesis (Fig. 5j). Specifically, BLCA, HNSC, and KIRC have decreased contributions from glutamate up-taken extracellularly; BRCA has increased contribution from glutamate up-taken extracellularly; COAD has unchanged contribution of glutamine from both sources; KICH has increased contributions from glutamate up-taken extracellularly but decreased contributions from glutamate converted from glutamine; KIRP has decreased contributions from glutamate up-taken extracellularly and converted from glutamine; and LUAD synthesize GSH using more glutamate that is converted from glutamine. We also observed that expression levels of rate-limiting enzymes involved in the synthesis of other anti-oxidation factors are up-regulated in eight types of cancer (Fig. 3g), suggesting other anti-oxidation factors, such as catalase [56] and thioredoxin [57], are also used to protect cancers from high levels of ROS.

### Correlations between ROS and glutamine as well as glutamate metabolisms

**Fig. 6:**
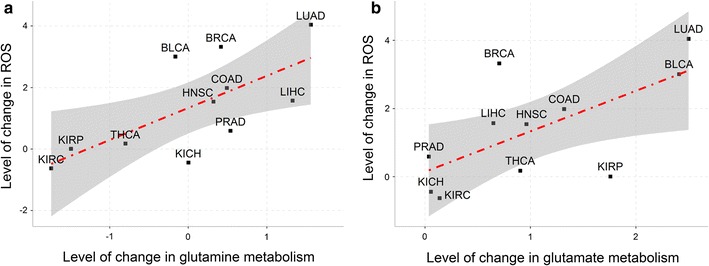
Strong correlations between the level of change in reactive oxygen species (ROS) and the levels of change in glutamine and glutamate metabolisms across 11 types of cancer. ROS level is correlated with glutamine metabolism (*r* = 0.685, *P* = 0.020) (**a**) and glutamate metabolism (*r* = 0.655, *P* = 0.029) (**b**). In each panel, the *X* axis represents the difference between the expression levels of glutamine/glutamate metabolism in cancer versus control tissues, and the *Y* axis represents the difference between the ROS levels in cancer versus control tissues. BLCA, bladder urothelial carcinoma; BRCA, breast invasive carcinoma; COAD, colon adenocarcinoma; HNSC, head and neck squamous cell carcinoma; KICH, kidney chromophobe; KIRC, kidney renal clear cell carcinoma; KIRP, kidney renal papillary cell carcinoma; LIHC, liver hepatocellular carcinoma; LUAD, lung adenocarcinoma; PRAD, prostate adenocarcinoma; THCA, thyroid carcinoma

We estimated the intracellular level of change in ROS in each of the 11 types of cancer by using genes involved in proteasome and observed a strong positive correlation between the estimated level of change in ROS and the level of change in glutamine metabolism, with a corresponding *r* of 0.686 and a *P* value of 0.020 (Fig. 6a), as well as that between the level of change in ROS and the level of change in glutamate metabolism, with a corresponding *r* of 0.655 and a *P* value of 0.029, across the 11 types of cancer (Fig. 6b). Specifically, the level of change in ROS represents difference between ROS levels in cancer versus control tissues, and the level of change in glutamine/glutamate metabolism represents the difference between the expression levels of glutamine/glutamate metabolism in cancer versus control tissues.

These correlations had been previously attributed to the anti-oxidation function of GSH synthesized from glutamate and indirectly from glutamine [15]. However, our data suggested that this explanation may not be absolutely correct since it can explain only the correlation between the ROS level and the glutamate metabolism.

Nucleotide synthesis is a major sink for glutamine [16, 19], which is supported by our data in the previous subsection. We had previously discovered that, when the cytosolic concentration of H_2_O_2_ is sufficiently high, Fenton reaction ${\text{H}}_{ 2} {\text{O}}_{ 2} + {\text{ Fe}}^{ 2+ } \to {\text{ Fe}}^{ 3+ } + {\text{ OH}}^{ - } + \;{\text{OH}} \cdot$ [58] may take place in all cancers [59]. If there is a rich supply of reducing elements nearby that can reduce Fe^3+^ to Fe^2+^, Fenton reactions would continue indefinitely, which would give rise to continuous production of ${\text{OH}} \cdot$, the most reactive molecule that human cells could produce [59]. Our previous study had revealed that this will lead to substantially increased proteasome assemblies and activities in cancer to degrade proteins damaged by ${\text{OH}} \cdot$ [59]. A recent study found that a reduced concentration of glutamine inhibits ubiquitin–proteasome activities [60], suggesting that glutamine is directly involved in such activities. Our previous research has revealed that the Fenton reaction-produced OH^−^ drives nucleotide synthesis, which requires glutamine. To rule out the possibility that our observed co-expression of glutamine and proteasome genes is caused by Fenton reaction-induced nucleotide synthesis, we re-assessed the co-expression between glutamine and proteasome genes under the condition when the nucleotide synthesis rate is constant. Specifically, rate-limiting enzymes of nucleotide synthesis from glutamine, phosphoribosyl pyrophosphate amidotransferase (PPAT) and carbamoyl-phosphate synthetase 2, aspartate transcarbamylase, and dihydroorotase (CAD) [61], were used to represent the level of nucleotide synthesis, and we selected samples with constant nucleotide synthesis level, then calculated the associations between glutamine and proteasome genes using this subset of samples. The correlation values across the 11 cancer types are summarized in Table 4, and the significance of all the correlations is with a *P* value no more than 0.05.

**Table 4:** Co-expression levels of PPAT and CAD with proteasome genes in 11 types of cancer

Cancer	Gene									
	PPAT					CAD				
	PSMD1	PSMD11	PSMD14	PSME3	PSME4	PSMB5	PSMD1	PSMD2	PSME3	PSME4
BLCA	0.26	0.37	0.37	0.38	0.23	0.26	0.33	0.48	0.29	0.24
BRCA	0.36	0.42	0.32	0.37	0.29			0.33		0.35
COAD		0.27	0.38	0.33					0.29	0.36
HNSC		0.33	0.24	0.36	0.33				0.29	0.37
KICH	0.36	0.33	0.25	0.43	0.47	0.27				
KIRC	0.26	0.38			0.29			0.22		0.37
KIRP	0.44	0.49	0.47	0.28	0.35		0.28	0.24		0.24
LIHC	0.24	0.27	0.25	0.45					0.43	
LUAD	0.33	0.48	0.46	0.47	0.42		0.3	0.36	0.32	0.47
PRAD		0.35		0.43	0.36			0.29	0.34	0.26
THCA		0.48		0.42	0.25			0.4		

PPAT, phosphoribosyl pyrophosphate amidotransferase; CAD, carbamoyl-Phosphate synthetase 2, aspartate transcarbamylase, and dihydroorotase; PSMD1, proteasome 26S subunit, non-ATPase 1; PSMD11, proteasome 26S subunit, non-ATPase 11; PSMD14, proteasome 26S subunit, non-ATPase 14; PSME3, proteasome activator subunit 3; PSME4, proteasome activator subunit 4; PSMB5, proteasome subunit beta 5; PSMD2, proteasome 26S subunit, non-ATPase 2

## Discussion

Through comparative analyses of gene expression data of 11 types of cancer, we computationally predicted how glutamine and glutamate contribute to cancer biology. Specifically, we observed that (i) the increased influx of glutamine in cancer is mainly due to up-regulated importers, whereas increased influx of glutamate is due to both increased conversion from glutamine and increased uptake, depending on specific types of cancer; (ii) glutamine and glutamate metabolisms are mostly increased in cancer, and the level of change in glutamine strongly correlates with the 5-year survival rate; (iii) our analyses in terms of the levels of statistical contributions by glutamine and/or glutamate to seven pathways reveal the following novel information: (1) glutamine generally does not contribute to purine synthesis in cancer except for BRCA, similarly not to pyridine synthesis except for KIRC; (2) glutamine generally does not contribute to ATP production in cancer; (3) the contribution to nucleotide synthesis by glutamine is minimal if any in cancer; (4) glutamine does not contribute to asparagine synthesis in cancer except for BLCA and LUAD; and (5) glutamate generally does not contribute to serine synthesis except for BLCA; and (iv) strong correlations between increased glutamine and glutamate metabolisms and increased ROS level suggest an anti-oxidation function of glutamine and glutamate.

Different from cell line-based studies, our analysis was conducted on gene expression data of cancer and control tissues. Hence, the analysis results offered a more accurate reflection of the functional roles played by glutamine and glutamate in cancer. In the meantime, tissue-based gene expression data are considerably more complex than cell line data, as the observed gene expression data have contributions from non-cancer cells, such as immune cells, stromal cells, and fat cells, which clearly raises an issue of how reliable the estimated results are, particularly when the percentage of cancer cells in different tissues may vary, in some cases substantially. Knowing this information, we have to note that the present study is limited by the complications of multiple types of cells in cancer tissues, because subtle changes in terms of differential expression may not be detectable using our present analyses of the tissue-based data. Hence up- or down-regulated genes should be considered to be substantially up- or down-regulated. To overcome this limitation, further studies to tease out the true expression of cancer cells in the tissue data are necessary, before we could detect more subtle changes within cancer cells.

## Abbreviations

BLCA: bladder urothelial carcinoma
BRCA: breast invasive carcinoma
COAD: colon adenocarcinoma
HNSC: head and neck squamous cell carcinoma
KICH: kidney chromophobe
KIRC: kidney renal clear cell carcinoma
KIRP: kidney renal papillary cell carcinoma
LIHC: liver hepatocellular carcinoma
LUAD: lung adenocarcinoma
PRAD: prostate adenocarcinoma
THCA: thyroid carcinoma

## References

[1] Vinnars E, Bergstom J, Furst P (1975). Influence of the postoperative state on the intracellular free amino acids in human muscle tissue. Ann Surg.

[2] Hammoudi N, Ahmed KBR, Garciaprieto C, Huang P (2011). Metabolic alterations in cancer cells and therapeutic implications. Chin J Cancer..

[3] Fuchs BC, Bode BP (2005). Amino acid transporters ASCT2 and LAT1 in cancer: partners in crime?. Semin Cancer Biol.

[4] Deberardinis RJ, Mancuso A, Daikhin E, Nissim I, Yudkoff M, Wehrli S (2008). Beyond aerobic glycolysis: Transformed cells can engage in glutamine metabolism that exceeds the requirement for protein and nucleotide synthesis. Proc Natl Acad Sci.

[5] Son J, Lyssiotis CA, Ying H, Wang X, Hua S, Ligorio M (2013). Glutamine supports pancreatic cancer growth through a KRAS-regulated metabolic pathway. Nature.

[6] Reitzer LJ, Wice BM, Kennell D (1979). Evidence that glutamine, not sugar, is the major energy source for cultured HeLa cells. J Biol Chem.

[7] Mehrmohamadi M, Liu X, Shestov AA, Locasale JW (2011). Characterization of the usage of the serine metabolic network in human cancer. Cell Rep..

[8] Tedeschi PM, Markert EK, Gounder M, Lin H, Dvorzhinski D, Dolfi SC (2013). Contribution of serine, folate and glycine metabolism to the ATP, NADPH and purine requirements of cancer cells. Cell Death Dis..

[9] Yoshida S, Kaibara A, Yamasaki K, Ishibashi N, Noake T, Kakegawa T (1995). Effect of glutamine supplementation on protein metabolism and glutathione in tumor-bearing rats. J Parenter Enteral Nutr.

[10] Todorova VK, Harms SA, Kaufmann Y, Luo SK, Luo KQ, Babb K (2004). Effect of dietary glutamine on tumor glutathione levels and apoptosis-related proteins in DMBA-induced breast cancer of rats. Breast Cancer Res Treat.

[11] Suzuki S, Tanaka T, Suyama K, Yokote K, Prives C, Tatsuno I. Phosphate activated glutaminase (GLS2), a novel p53-inducible regulator of glutamine metabolism and reactive oxygen species. Endocr Rev. 2010;31(3):7461–6. 10.1073/pnas.1002459107 PMC2867754 20351271

[12] Sodi VL, Khaku S, Krutilina R, Schwab LP, Vocadlo DJ, Seagroves TN (2015). mTOR/MYC axis regulates O-GlcNAc transferase (OGT) expression and O-GlcNAcylation in breast cancer. Mol Cancer Res..

[13] Iozzo RV, Clark CC (1987). Modulation of heparan sulfate synthesis of human colon carcinoma cells by 6-diazo-5-oxo-l-norleucine (DON), a glutamine analog. Fed Proc Fed Am Soc Exp Biol (United States)..

[14] Yang C, Sudderth J, Dang T, Bachoo RG, Mcdonald JG, Deberardinis RJ (2009). Glioblastoma cells require glutamate dehydrogenase to survive impairments of glucose metabolism or Akt signaling. Can Res.

[15] Koglin N, Mueller A, Berndt M, Schmitt-Willich H, Toschi L, Stephens AW (2011). Specific PET imaging of xC-transporter activity using a ^18^F-labeled glutamate derivative reveals a dominant pathway in tumor metabolism. Clin Cancer Res.

[16] Wise DR, Thompson CB (2010). Glutamine addiction: a new therapeutic target in cancer. Trends Biochem Sci.

[17] Souba WW (1993). Glutamine and cancer. Ann Surg.

[18] Hensley CT, Wasti AT, Deberardinis RJ (2013). Glutamine and cancer: cell biology, physiology, and clinical opportunities. J Clin Invest..

[19] Deberardinis RJ, Cheng T (2009). Q’s next: the diverse functions of glutamine in metabolism, cell biology and cancer. J Acid Emerg Med.

[20] Tomczak K, Czerwinska P, Wiznerowicz M (2015). The cancer genome atlas (TCGA): an immeasurable source of knowledge. Contemp Oncol..

[21] Yli-Hietanen J, Ylipää A, Yli-Harja O (2015). Cancer research in the era of next-generation sequencing and big data calls for intelligent modeling. Chin J Cancer..

[22] Hampton T (2006). Cancer genome atlas. J Am Med Assoc..

[23] Ritchie ME, Phipson B, Wu D, Hu Y, Law CW, Shi W (2015). Limma powers differential expression analyses for RNA-sequencing and microarray studies. Nucleic Acids Res..

[24] S KPFR. LIII (2010). On lines and planes of closest fit to systems of points in space. Philos Mag Ser 1..

[25] Tabachnick BG, Fidell LS. Using multivariate statistics. 6th ed. London: Pearson; 2013.

[26] Van GM, Wang Q, Nagarajah R, Marshall AD, Thoeng A, Gao D (2015). ASCT2/SLC1A5 controls glutamine uptake and tumour growth in triple-negative basal-like breast cancer. Oncogene.

[27] Liu Y, Liu Y, An H, Yuan C, Zhang W, Zhu Y (2015). High expression of Solute Carrier Family 1, member 5 (SLC1A5) is associated with poor prognosis in clear-cell renal cell carcinoma. Sci Rep..

[28] Hassanein M, Hoeksema MD, Shiota M, Qian J, Harris BK, Chen H (2013). SLC1A5 mediates glutamine transport required for lung cancer cell growth and survival. Clin Cancer Res Off J Am Assoc Cancer Res..

[29] Hassanein M, Qian J, Hoeksema MD, Wang J, Jacobovitz M, Ji X (2015). Targeting SLC1a5-mediated glutamine dependence in non-small cell lung cancer. Int J Cancer..

[30] Huang F, Zhao Y, Zhao J, Wu S, Jiang Y, Ma H (2014). Upregulated SLC1A5 promotes cell growth and survival in colorectal cancer. Int J Clin Exp Pathol..

[31] Kondoh N, Imazeki N, Arai M, Hada A, Hatsuse K, Matsuo H (2007). Activation of a system A amino acid transporter, ATA1/SLC38A1, in human hepatocellular carcinoma and preneoplastic liver tissues. Int J Oncol.

[32] Wang K, Cao F, Fang W, Hu Y, Chen Y, Ding H (2013). Activation of SNAT1/SLC38A1 in human breast cancer: correlation with p-Akt overexpression. Bmc Cancer..

[33] Yu WL, Cong WM, Zhang Y, Chen Y, Wang F, Yu G (2011). Overexpression of ATA1/SLC38A1 predicts future recurrence and death in chinese patients with hilar cholangiocarcinoma. J Surg Res.

[34] Xie J, Li P, Gao HF, Qian JX, Yuan LY, Wang JJ (2014). Overexpression of SLC38A1 is associated with poorer prognosis in Chinese patients with gastric cancer. Bmc Gastroenterol..

[35] Oh RS, Pan WC, Yalcin A, Zhang H, Guilarte TR, Hotamisligil GS (2012). Functional RNA interference (RNAi) screen identifies system a neutral amino acid transporter 2 (SNAT2) as a mediator of arsenic-induced endoplasmic reticulum stress. J Biol Chem.

[36] Chaudhry FA, Reimer RJ, Krizaj D, Barber D, Stormmathisen J, Copenhagen DR (1999). Molecular analysis of system N suggests novel physiological roles in nitrogen metabolism and synaptic transmission. Cell.

[37] Nakanishi T, Sugawara M, Huang W, Martindale RG, Leibach FH, Ganapathy ME (2001). Structure, function, and tissue expression pattern of human SN2, a subtype of the amino acid transport system N. Biochem Biophys Res Commun.

[38] Ap VDH, Jing J, Wooster RF, Bachman KE (2012). Analysis of glutamine dependency in non-small cell lung cancer: GLS1 splice variant GAC is essential for cancer cell growth. Cancer Biol Ther.

[39] Altman BJ, Stine ZE, Dang CV (2016). From Krebs to clinic: glutamine metabolism to cancer therapy. Nat Rev Cancer.

[40] Ambs A, Warren JL, Bellizzi KM, Topor M, Haffer SC, Clauser SB (2008). Overview of the SEER—medicare health outcomes survey linked dataset. Health Care Financ Rev..

[41] Lu W, Pelicano HP (2010). Cancer metabolism: is glutamine sweeter than glucose?. Cancer Cell.

[42] Hakomori S (2002). Glycosylation defining cancer malignancy: new wine in an old bottle. Proc Natl Acad Sci USA.

[43] Pinho SS, Reis CA (2015). Glycosylation in cancer: mechanisms and clinical implications. Nat Rev Cancer.

[44] Roseman S (2001). Reflections on glycobiology. J Biol Chem.

[45] Krall AS, Xu S, Graeber TG, Braas D, Christofk HR (2016). Asparagine promotes cancer cell proliferation through use as an amino acid exchange factor. Nat Commun..

[46] Phang JM, Donald SP, Pandhare J, Liu Y (2008). The metabolism of proline, a stress substrate, modulates carcinogenic pathways. Amino Acids.

[47] Barbato DL, Aquilano K, Baldelli S, Cannata SM, Bernardini S, Rotilio G (2014). Proline oxidase|[ndash]|adipose triglyceride lipase pathway restrains adipose cell death and tissue inflammation. Cell Death Differ.

[48] Phang JM, Liu W, Hancock CN, Fischer JW (2015). Proline metabolism and cancer: emerging links to glutamine and collagen. Curr Opin Clin Nutr Metab Care..

[49] Piskac-Collier AL, Monroy C, Lopez MS, Cortes A, Etzel CJ, Greisinger AJ (2011). Variants in folate pathway genes as modulators of genetic instability and lung cancer risk. Genes Chromosom Cancer.

[50] Zhang WC, Shyhchang N, Yang H, Rai A, Umashankar S, Ma S (2012). Glycine decarboxylase activity drives non-small cell lung cancer tumor-initiating cells and tumorigenesis. Cell.

[51] Bhattacharyya S, Saha S, Giri K, Lanza IR, Nair KS, Jennings NB (2013). Cystathionine beta-synthase (CBS) contributes to advanced ovarian cancer progression and drug resistance. PLoS ONE..

[52] Sen S, Kawahara B, Gupta D, Tsai R, Khachatryan M, Roychowdhuri S (2015). Role of cystathionine β-synthase in human breast Cancer. Free Radic Biol Med.

[53] Maddocks OD, Labuschagne CF, Adams PD, Vousden KH (2016). Serine metabolism supports the methionine cycle and DNA/RNA Methylation through de novo ATP synthesis in cancer cells. Mol Cell.

[54] Amelio I, Markert EK, Rufini A, Antonov A, Sayan BS, Tucci P (2014). p73 regulates serine biosynthesis in cancer. Oncogene.

[55] Traverso N, Ricciarelli R, Nitti M, Marengo B, Furfaro AL, Pronzato MA (2013). Role of glutathione in cancer progression and chemoresistance. Oxid Med Cell Longev..

[56] Bechtel W, Bauer G (2009). Catalase protects tumor cells from apoptosis induction by intercellular ROS signaling. Anticancer Res.

[57] Lincoln DT, Emadi EMA, Tonissen KF, Clarke FM (2003). The thioredoxin–thioredoxin reductase system: over-expression in human cancer. Anticancer Res.

[58] Fenton HJH. LXXIII.—Oxidation of tartaric acid in presence of iron. J Chem Soc Trans. 1894;65:899–910.

[59] Sun H, Zhang C, Dong N, Sheng T, Xu Y. Fenton reactions drive nucleotide and ATP syntheses in cancer, and implications. 2017 (Under review). 10.1093/jmcb/mjy039 PMC6231523 30016460

[60] Zellner M, Gerner C, Eliasen MM, Wurm S, Pollheimer J, Spittler A (2003). Glutamine starvation of monocytes inhibits the ubiquitin-proteasome proteolytic pathway. Biochem Biophys Acta.

[61] Zhao M, Chen X, Gao G, Tao L, Wei L (2009). RLEdb: a database of rate-limiting enzymes and their regulation in human, rat, mouse, yeast and *E. coli*. Cell Res.

